# Safety, Pharmacokinetic and Pharmacodynamic Evaluation of Teverelix for the Treatment of Hormone-Sensitive Advanced Prostate Cancer: Phase 2 Loading-Dose-Finding Studies

**DOI:** 10.3390/medicina59040681

**Published:** 2023-03-29

**Authors:** Carol M. MacLean, Albertas Ulys, Feliksas Jankevičius, Žilvinas Saladžinskas, Steve van Os, Finn Larsen

**Affiliations:** 1 Antev Ltd., London W1S 1DN, UK; 2 National Cancer Institute, 08406 Vilnius, Lithuania; 3 Faculty of Medicine, Vilnius University, Ciurlionio 21, LT-03101 Vilnius, Lithuania; 4 Department of Surgery, Medical Academy, Hospital of Lithuanian University of Health Sciences, 50103 Kaunas, Lithuania

**Keywords:** androgen deprivation therapy, cardiovascular risk, castration, gonadotropin-releasing hormone agonist, luteinizing hormone-releasing hormone agonists, gonadotropin-releasing hormone antagonist, prostate cancer

## Abstract

*Background and objectives:* Teverelix drug product (DP) is a gonadotropin-releasing hormone antagonist in development for the treatment of patients with prostate cancer in whom androgen deprivation therapy is indicated. The aim of this paper is to present the results of five Phase 2 studies that assessed the pharmacokinetics, pharmacodynamics, efficacy and safety of different loading dose regimens of teverelix DP. *Methods:* Five single-arm, uncontrolled clinical trials were conducted in patients with advanced prostate cancer. The five different loading dose regimens of teverelix DP tested were (a) a single 90 mg subcutaneous (SC) injection of teverelix DP given on 3 consecutive days (Days 0, 1 and 2); (b) a single 90 mg intramuscular (IM) injection of teverelix DP given 7 days apart (Days 0 and 7); (c) a single 120 mg SC injection of teverelix DP given on 2 consecutive days (Days 0 and 1); (d) 2 × 60 mg SC injections of teverelix DP given on 3 consecutive days (Days 0, 1 and 2), and (e) 2 × 90 mg SC injections of teverelix DP given on 3 consecutive days (Days 0, 1 and 2). The primary efficacy parameter was the duration of action of an initial loading dose regimen in terms of suppression of testosterone to below the castration level (0.5 ng/mL). *Results:* Eighty-two patients were treated with teverelix DP. Two regimens (90 mg and 180 mg SC on 3 consecutive days) had a mean duration of castration of 55.32 days and 68.95 days with >90% of patients having testosterone levels < 0.5 ng/mL at Day 28. The mean onset of castration for the SC regimens ranged from 1.10 to 1.77 days, while it was slower (2.4 days) with IM administration. The most common adverse event (AE) was injection site reaction. No AEs of severe intensity were reported. *Conclusions:* Teverelix DP is safe and well tolerated. Castrate levels of testosterone can be rapidly achieved following the subcutaneous injection of teverelix DP on 3 consecutive days. Streamlining of the administration of the loading dose and identifying a suitable maintenance dose will be investigated in future trials.

## 1. Introduction

Androgen deprivation therapy (ADT) is the cornerstone of treatment for hormone-sensitive advanced prostate cancer, which is usually achieved with the use of gonadotrophic-releasing hormone (GnRH) analogs [1,2]. Numerous GnRH agonists have been available since the 1980s, and they continue to dominate the market despite some limitations. GnRH agonists mimic GnRH, and by binding to GnRH receptors in the pituitary gland, they ultimately lead to a decrease in the release of luteinizing hormone (LH), follicle-stimulating hormone (FSH) and, in males, testosterone [3]. However, initially, they stimulate GnRH receptors, resulting in a significant increase in LH and FSH and consequently a surge in testosterone levels that may exacerbate disease and symptoms (disease flare) and leads to a slow onset of action (3 to 4 weeks) [3]. It is also of note that GnRH agonists may be associated with an increased risk of cardiovascular disease (CVD) and of diabetes [3,4,5,6].

GnRH antagonists, on the other hand, bind and block the action of GnRH receptors directly, with a rapid decrease in LSH, FSH and testosterone, without the delay and testosterone flare that occurs with GnRH agonists [3]. A small number of GnRH antagonists have been licensed for advanced prostate cancer. The first GnRH antagonist to be approved was plenaxis (abarelix) in the US in 2003 [7]. Abarelix, however, has been associated with an increased risk of immediate onset systemic allergic reactions requiring an extensive risk management program [8,9], which may have contributed to a commercial decision by the company to discontinue its use in some countries, including the US. Degarelix was approved in 2008, but despite its efficacy, it is still not widely used in clinical practice, which is possibly due to the frequency of injections required (every month compared to 1-, 3-, 6- and 12-month formulations with GnRH agonists) and a high (~40%) incidence of injection-site reactions [9,10]. The first oral GnRH antagonist, relugolix, was approved in 2020 [11]. Overall, there is still a need for additional GnRH antagonists with good efficacy, safety and tolerability profiles, and administration schedules suited to patients and healthcare resources. Furthermore, although still not definitive, there is some evidence that GnRH antagonists may have a better CV safety profile than GnRH agonists [11,12,13,14,15], which could lead to a paradigm shift in treatment options, especially as a large proportion of men initiating treatment for prostate cancer have pre-existing CV events or are at high risk for CV events [14,16,17], further demonstrating the unmet need for new GnRH antagonists.

Teverelix drug product (DP) is a decapeptide GnRH antagonist characterized by relatively good water solubility, little in vitro aggregation, and low histamine-releasing potency, with a dose that produces the half-maximal response [18]. Upon reconstitution, a fixed amount of trifluoroacetate (TFA) can be added to the teverelix base, achieving an exact and desired molar ratio to obtain a microcrystalline aqueous teverelix–TFA suspension at a concentration of 75 mg/mL [18]. This enables the parenteral (subcutaneous [SC] or intramuscular (IM)) administration of a large amount of peptide in a relatively small volume. The injection of teverelix DP via either the SC or IM route has been shown to result in a rapid (within 1–2 days) suppression of LH, FSH, testosterone (in males) [19,20] and E_2_ (in females) [21].

Initial trials with teverelix DP have focused on identifying an effective loading dose and establishing its duration of action [19,20]. In prostate cancer, where the goal is to attain and maintain profound castrate levels of testosterone, the challenge is to deliver sufficiently high initial amounts of peptide into the circulation to compete against endogenous GnRH and occupy pituitary GnRH receptors 100%. Following the attainment of castration with an initial loading dose of GnRH antagonist, the maintenance of castration can be achieved with a lower maintenance dose of GnRH antagonist [22].

Here, we describe the results of five Phase 2 clinical trials that were designed to evaluate the pharmacokinetics, pharmacodynamics, efficacy and safety of six different loading dose regimens (doses, schedules and administration methods (SC and IM)) of teverelix DP in patients with advanced prostate cancer.

## 2. Materials and Methods

### 2.1. Study Design, Conduct and Treatment

Five clinical trials were conducted at investigational sites in Lithuania and Latvia between 2004 and 2007 with each trial testing a different loading dose and/or regimen of teverelix DP in patients with advanced, hormone-sensitive prostate cancer (Supplemental Table S1). The studies were all open-label, uncontrolled trials that were either single-center (2 trials evaluating 1 regimen in each trial) or multi-center (2 trials evaluating 1 regimen and 1 randomized trial evaluating 2 different loading doses).

All studies were conducted in accordance with the current version of the Declaration of Helsinki and with favorable opinion from the competent authority and independent ethics committee for each investigational site (Supplemental Table S1). Four of the five trials were registered with the European Union Drug Regulating Authorities Clinical Trials Database under the EudraCT reference numbers: 2004-001648-64 for trial EP-24332T-A014; 2005-002100-42 for trial ARD-0301-003; 2005-005742-39 for trial ARD-0301-008 and 2006-004572-13 for trial ARD-0301-010 (Supplemental Table S1). Trial EP-24332T-A013 pre-dated the requirement for registration. The trials were conducted between 2004 and 2007; however, the trials’ sponsor Ardana Bioscience Ltd. subsequently went into administration in 2009 before the results were published. Antev Ltd., who acquired the intellectual property for teverelix in 2014, re-started the clinical development program in 2019 based on the continued unmet need for a new GnRH antagonist in men with advanced prostate cancer.

**Figure 1:**
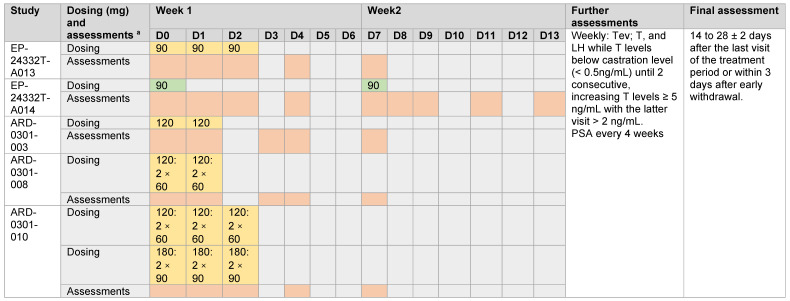
Teverelix DP dose and regimen and evaluation days in the Phase 2 clinical trials. Yellow box = subcutaneous (SC) injection; green box = intramuscular (IM) injection; orange box = assessment days for teverelix, testosterone and luteinizing hormone. D = day; LH = luteinizing hormone; PSA = prostate-specific antigen; Tev = teverelix; T = testosterone. ^a^ Tev, T, LH and PSA were measured during screening and at baseline prior to dosing. Teverelix levels were assessed at 1, 2, 4, 6 and 10 and 24 h after dosing and on days specified. PSA was measured at screening, Day 7 and Weeks 2 (ARD-0301-003, ARD-0301-008 and ARD-0301-010 only), 4, 8 and 12 and the final assessment.

Teverelix DP was supplied as a freeze-dried powder without any excipient, which was reconstituted with 5% mannitol (*w*/*v*) in water for injection to a final concentration of 75 mg/mL. Injections were given by SC administration into the lower abdominal wall or IM administration into the buttock. Patients received treatment according to the dose and regimen shown in Figure 1, initially for 3 weeks and then until their testosterone levels increased for 2 consecutive study visits (above 2 ng/mL for the 2nd consecutive visit) (Figure 1).

### 2.2. Patients

The eligibility criteria were the same for all the trials:

Inclusion criteria

- Histologically proven adenocarcinoma of the prostate;
- Androgen deprivation therapy suitable (advanced prostate cancer, i.e., with local invasion or/and metastasis);
- Signed written informed consent.

Exclusion criteria

- Liver or renal function tests (ASAT/SGOT, ALAT/SGPT, total bilirubin, creatinine) exceeding twice the upper limit of the normal range, unless the elevation is attributed to hepatic metastasis;
- Any contraindication to the use of Teverelix DP;
- Life expectancy of less than 1 year;
- Baseline testosterone value below 2.31 ng/mL;
- Bilateral orchidectomy;
- Pre-existing hormone therapy or planned concomitant use of androgen deprivation therapy with any agent other than the investigational drug;
- Neurological, psychiatric disease, drug or alcohol abuse which could interfere with the subject’s proper compliance;
- Evidence of concurrent malignancy;
- Exposure to another investigational agent within the last month;
- Lack of ability or willingness to give informed consent;
- Anticipated non-availability for study visits/procedures.

The subject populations were male patients with histologically proven adenocarcinoma of the prostate and for whom ADT was considered suitable (advanced prostate cancer, i.e., with local invasion and/or metastasis as staged by the Tumor, Nodes, Metastisis (TNM) scale (see below)). Patient demographics are detailed in Supplemental Table S1. Patients were not eligible for the trials if they had: a life expectancy of less than 1 year, a bilateral orchidectomy, pre-existing hormone therapy or planned concomitant use of ADT, a baseline testosterone value below 2.31 ng/mL, or abnormal liver or renal function tests (twice the upper limit of the normal range).

The TNM system is a way of staging prostate cancer. It stands for Tumor, Node, Metastasis. Tumor (T) describes the size or area of the cancer. There are 4 main T stages of prostate cancer–T1 to T4.

T1 means the cancer is too small to be seen on a scan or felt during an examination of the prostate.

T2 means the cancer is completely inside the prostate gland.

T3 means the cancer has broken through the capsule of the prostate gland.

T4 means the cancer has spread into other body organs nearby, such as the back passage, bladder, or the pelvic wall.

Node (N) describes whether the cancer has spread to the lymph nodes with N0 meaning that the nearby lymph nodes do not contain cancer cells and N1 means there are cancer cells in the lymph nodes near the prostate. NX means that the cancer in nearby lymph nodes cannot be measured.

Metastasis (M) describes whether the cancer has spread to a different part of the body, and there are two M stages: M0 and M1. M0 means the cancer has not spread to other parts of the body. M1 means the cancer has spread to other parts of the body outside the pelvis.

### 2.3. Outcomes and Assessments

The primary objective of all the trials was to assess the duration of action of an initial loading dose regimen of teverelix DP in terms of suppression of testosterone to below the castration level (0.5 ng/mL). The secondary objectives were: to assess the pharmacodynamics of teverelix DP in terms of its ability to suppress and maintain plasma testosterone levels below the castration level after 3 weeks of treatment (<0.5 ng/mL until 2 consecutive, increasing testosterone levels above the castration level with the latter recording above 2 ng/mL); to assess the effects on LH and prostate-specific antigen (PSA); and to assess the safety of teverelix DP. The safety evaluation included adverse events (AEs), clinical laboratory tests, vital signs, physical examinations. AEs were coded using the Medical Dictionary for Regulatory Activities (MedDRA v7.0, Herndon, VA, USA). In addition, a formal injection site inspection was performed in 3 trials (ARD-0301-003, ARD-0301-008 and ARD-0301-010) to assess local tolerability, i.e., redness, swelling and induration (size/diameter, nature and firmness), itching and pain/tenderness. A transrectal ultrasound (TRUS) to assess total prostatic volume and the volume of peripheral and transitional zones of the prostate was conducted in the same three trials.

Serum total testosterone and luteinizing hormone (LH) were measured using validated assays at MDS Pharma Services, Grossmoorbogen 25, 21,079 Hamburg, Germany. Total testosterone and LH was measured by double-antibody RIA kits from MP Biochemicals (formerly ICN Biochemicals, Santa Ana, CA, USA) after extraction of the serum with hexane and ethyl acetate using a gamma-counter Berthold 2104 with its own evaluation software. The assay kits were already validated by the producer for the standard use in human serum or plasma. Re-validation of the testosterone assay was completed by MDS Pharma Services (King of Prussia, PA, USA). The lower limit of quantification (LLoQ) for testosterone was reported as 0.0259 ng/mL. For LH, the LLoQ was 0.3 IU/L. All analyses and reporting were completed in accordance with Good Laboratory Practice (GLP).

Teverelix levels were analyzed in plasma samples by HFL Ltd., Fordham, UK or by Professor Huy Ong’s laboratory at the University of Montreal, Montreal, QC, Canada. PSA measurements were performed at local laboratories. The assessment days are shown in Figure 1.

### 2.4. Statistical Analysis

No formal sample size calculations or between-group comparisons were performed for these explorative Phase 2 studies that were designed to obtain additional knowledge to elucidate further Phase 2/3 studies. The primary analysis was performed for the intent-to-treat (ITT) dataset, which included all patients who received at least one dose of study drug and at least one testosterone measurement after first administration of the study drug. The safety evaluation was based on all patients who received at least one dose of study medication. Descriptive statistics were conducted for all study variables. Categorical data were presented using counts and percentages, whilst continuous variables were presented using the mean, standard deviation (SD), standard error of the mean (SEM), median and range.

## 3. Results

### 3.1. Patients

Overall, 82 patients were enrolled and received treatment in the 5 clinical trials (*n* = 14, 14, 8, 8 and 38, respectively) (Table 1). The baseline patient characteristics are shown in Table 1. All patients were male and all were Caucasian. The majority of patients across the trials had locally advanced disease (i.e., T3/4, NX or N0, and M0, or N1 and M0) and a Gleason score of ≤7.

### 3.2. Efficacy, Pharmacokinetic and Pharmacodynamic Evaluations

The mean onset of castration for the SC regimens ranged from 1.10 days (120 mg SC for 2 days) to 1.77 days (90 mg for 2 days), while it was 2.4 days with 90 mg IM administration. The mean duration of castration across the studies ranged from 32.45 days (range: 0–6 weeks for 120 mg SC for 2 consecutive days) to 68.95 days (range 5–21 weeks for 180 mg SC for 3 consecutive days) (Table 2). In total, 3 (out of 82) subjects were not castrated at any time point following injection of teverelix DP. The 3 groups that had dosing on 3 consecutive days had the longest mean duration of castration: 55.32 days for 90 mg SC for 3 days; 60 days for 120 mg SC for 3 days and 68.95 days for 180 mg SC for 3 days. In trials EP-24332T-A013 (90 mg SC for 3 consecutive days) and ARD-0301-010 (180 mg SC for 3 consecutive days), the castration rate was >90% at Day 28 (100% and 95%, respectively) (Table 2).

**Figure 2:**
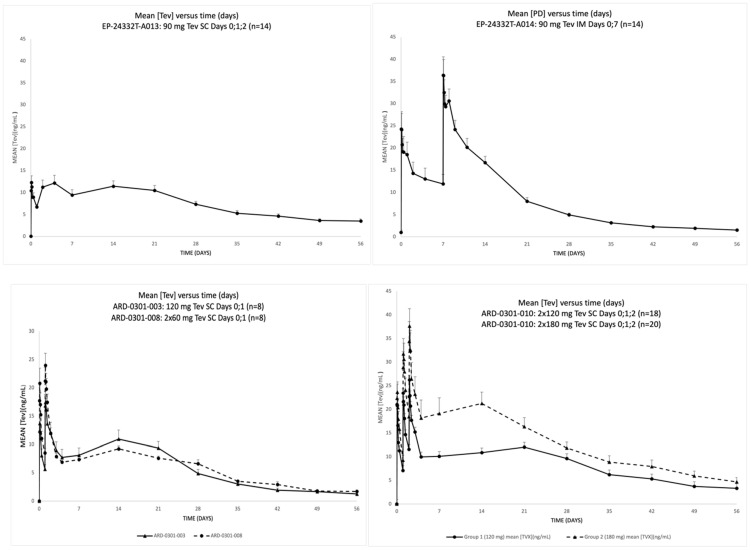
Mean teverelix DP concentration (ng/mL)(+SEM) vs. time (days). IM = intramuscular; SC = subcutaneous.

**Figure 3:**
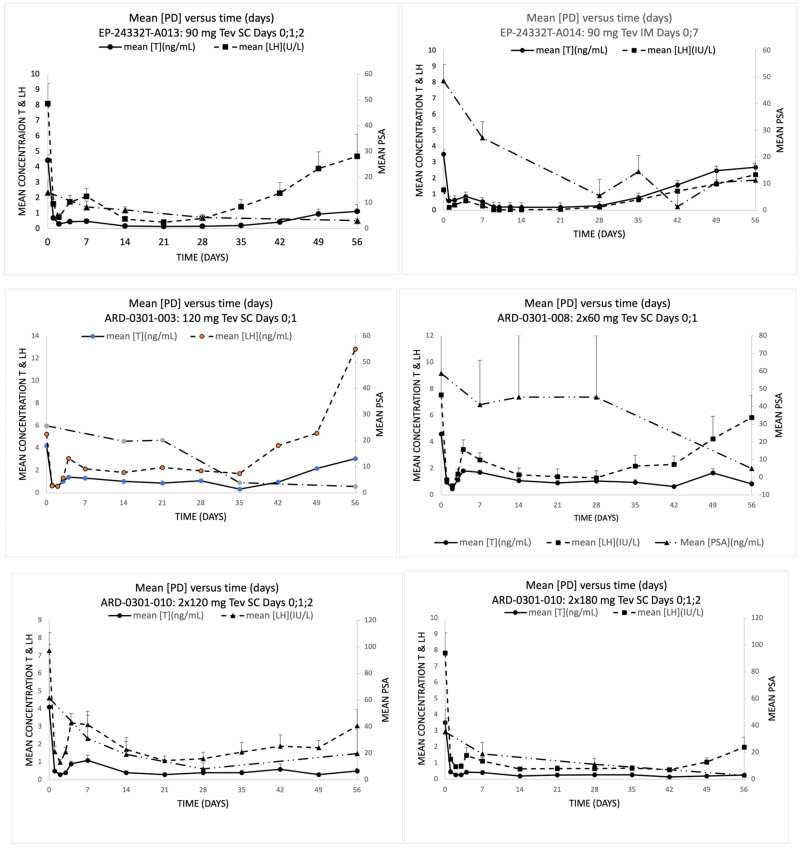
Mean total testosterone (ng/mL), luteinizing hormone (IU/L) and PSA (ng/mL)(+SEM) vs. time (days). IM = intramuscular; LH = luteinizing hormone; PD, pharmacodynamics; PSA = prostate-specific antigen; SC = subcutaneous; T = testosterone.

The mean pharmacokinetic (PK) results (teverelix DP concentrations (ng/mL) +SEM) for the studies are presented in Figure 2, the results of the PK parameters are shown in Supplemental Table S2, and the mean pharmacodynamic (PD) results (+SEM) (total testosterone, LH and PSA concentrations) are presented in Figure 3. SC administration resulted in rapid dose-dependent increases in teverelix DP after the first dose, which generally increased further with subsequent administrations. This was accompanied by rapid decreases in testosterone, LH and PSA. Escape from castration followed a steady decrease in plasma teverelix DP concentration, with the 3 groups that had SC dosing on 3 consecutive days having the longest steady state of teverelix DP. IM administration with one dose over 2 weeks resulted in a slower decline of teverelix DP levels.

### 3.3. Safety and Tolerability

Overall, the 82 patients received 232 injections (16 via IM administration; 216 via SC administration). The most common AEs were injection site reactions (*n* = 38/82, 46.3%), hot flushes (*n* = 18/82, 22%), and cystitis (*n* = 5/82, 6.1%) (Table 3). One serious AE was reported (renal cell carcinoma that was considered not related to the study drug), and no AEs of severe intensity or serious unexpected serious adverse reactions (SUSARs) were reported. No clinically significant changes in laboratory parameters, vital signs, ECGs or body weight were reported in any of the trials. From the formal injection site inspections, reactions were generally mild, with induration being the most common reaction (Supplemental Table S3).

For the transrectal ultrasound (TRUS) assessment in clinical trial ARD-0301-008, a statistically significant mean change from baseline to the final assessment of −10.3 (95% CI: −19.9, −0.6) cm^3^ and −4.0 (−7.0, −1.0) cm^3^ was recorded for total prostatic volume and transitional zone volume, respectively; a non-statistically significant mean change from baseline of −6.3 (−13.5, 1.0) cm^3^ was recorded for peripheral zone volume. In clinical trial ARD-0301-010, a dose-dependent mean change from baseline to the final assessment visit was recorded for total prostatic volume (−6.46 [95% CI: −9.566, −3.354] and −13.62 [−20.623, −6.617] cm^3^ for group 1 and group 2, respectively), for transitional zone volume (−1.12 [−3.489, 1.249] and −3.46 [−5.398, −1.522] cm^3^, respectively), and for peripheral zone volume (−5.44 [7.533, −3.347] and −10.61 [−16.878, −4.432] cm^3^, respectively). There were no significant changes in clinical trial ARD-0301-003.

## 4. Discussion

Prostate cancer is the second most common cause of cancer-related deaths in men, representing a major source of morbidity and mortality. Androgen deprivation therapy (ADT) is the primary treatment for patients with advanced prostate cancer at disease presentation, which can be achieved either with surgical or chemical castration [23]. Both GnRH agonists and GnRH antagonists act as ADT, but they have different mechanisms of action. GnRH agonists work by initially increasing the production of testosterone, which is followed by a decrease in testosterone levels. GnRH antagonists, on the other hand, directly block the production of testosterone [3,23]. Data from clinical trials [24] and meta-analyses of clinical trials [25] suggest GnRH antagonists achieved improved PSA progression-free survival, overall survival, joint-related symptoms, urinary tract infection events and musculoskeletal events compared with GnRH agonists.

In the United States, cardiovascular disease is the main cause of death for prostate cancer patients, and patients with prostate cancer have a higher risk of death from non-cancer causes than the general population [26]. ADT is associated with a number of cardiometabolic side effects including decreased insulin sensitivity, changes in lipid profile, and an increased risk of thromboembolic and cerebrovascular events [27]. The risk seems to be highest in elderly patients who have had recent cardiovascular events before starting ADT [27]. According to a Scientific Statement issued by the American Heart Association in April 2021 [6], evidence suggests that there are differences in CV outcomes between GnRH agonists and antagonists. An open-label phase 2 study randomized men to treatment with a GnRH agonist versus a GnRH antagonist and included new cardiovascular events as a secondary end point [11]. In this trial, major cardiovascular or cerebrovascular events occurred among 20% of patients receiving a GnRH agonist versus 3% of those receiving a GnRH antagonist (*p* = 0.013). Similarly, a recently published phase 3 trial randomized patients to treatment with leuprolide (GnRH agonist) or relugolix (GnRH antagonist) and included cardiovascular outcomes as a secondary end point. Major cardiovascular events occurred among 6.2% of patients receiving the GnRH agonist versus 2.9% of patients receiving a GnRH antagonist within the first 12 months of treatment (HR 0.46 [95% CI, 0.24–0.88]) [11].

While the Phase 2 trials reported in this paper were first conducted several years ago, little progress has been made during that time in terms of injectable GnRH antagonists. Since degarelix was approved in 2008, there have been no new injectable GnRH antagonists. Data from mouse model studies suggest that the increased CV risk with GnRH agonists compared to GnRH antagonists may be attributable to FSH levels coupled with T-lymphocyte GnRH receptor activation [28,29]. As FSH is involved in augmenting fat and lipid storage and may cause a disruption of vascular integrity and unstable plaques, the activation of GnRH receptors on T-lymphocytes can result in unstable plaques. Both of these features—if confirmed in prospective randomized clinical trials—could provide the explanation for the superior CV safety expected with GnRH antagonists.

Therefore, the development of teverelix DP is still relevant and the development of teverelix DP is now continuing with a new sponsor with a focus on its CV safety compared to GnRH agonists and with the aim of an improved administration schedule (longer duration of action requiring fewer administrations) and local tolerability profile than degarelix.

The data from these five Phase 2 clinical studies adds to the evidence from a Phase 1 clinical study that was conducted in older, healthy male subjects, showing that teverelix DP has a good safety profile and is well tolerated [18]. In the Phase 1 study, the maximum concentration and exposure generally increased in proportion to the various doses evaluated (i.e., 60, 90 and 120 mg SC single doses), while reductions in LH, FSH and testosterone were more prolonged with an IM administration (90 mg) compared an SC administration at the same dose [18]. Here, we found that the longest mean duration of castration was observed after SC dosing for 3 consecutive days, and that IM administration had a delayed onset of castration compared to SC administration. Overall, the effective loading doses of teverelix DP, i.e., those achieving a >90% castration rate at Day 28, were identified in two of the five trials in patients receiving 90 mg SC and 180 mg SC for 3 consecutive days (trials EP-24332T-A013 and ARD-0301-010).

The most commonly reported AE In the studies where teverelix DP is administered via SC administration was an injection site reaction, particularly injection site induration, generally of a mild intensity. In all trials, an injection site inspection was performed at every study visit post-administration (either as a formal safety endpoint in three trials or as part of analyzing AEs in two trials), and it was not up to the trial subjects to report any reactions experienced as was the case in the pivotal degarelix clinical trial [10]. Injection site induration is a feature of the physicochemical properties of teverelix DP in that it forms a depot upon SC injection (most likely a gel-like depot). This lends the product its slow-release properties, but it is mild and not bothersome to the patients. As teverelix DP is released into the circulation, the induration reduces in size and invariably disappears over time. Due to the different milieu following IM injection—where there is a high degree of vascularization and less adipose tissue—induration is not an issue. Indeed, this different milieu explains the different PK profiles (late phase Tmax and Cmax) observed following SC versus IM administration [18].

The effective loading doses of teverelix DP required injections to be administered on 3 consecutive days, which is inconvenient for the patients and may have implications for healthcare costs and resource utilization. Ideally, the loading dose would be administered at a single clinic visit, and data from this study have laid the foundations for such a regimen.

An ongoing Phase 2B clinical trial (NCT04693507) with teverelix DP is testing initial loading doses of 120 mg SC plus 120 mg IM injections and 180 mg SC plus 180 mg IM injections—administered on Day 0—followed by maintenance doses of teverelix DP 120 mg SC or 180 mg SC at Day 42 and every 6 weeks up to Day 168. The establishment of an effective dosing regimen of teverelix DP in prostate cancer patients will facilitate the conduct of a Phase 3 trial in advanced prostate cancer patients at increased CV risk to test the potential CV superiority of teverelix DP versus leuprolide. The development of an ADT agent with improved CV safety will meet an unmet clinical need in this patient population.

## 5. Conclusions

There is an unmet clinical need for a GnRH antagonist that has improved local tolerability and a sustained duration of action. Teverelix DP has the potential to address both of these points. Good local tolerability has been evidenced in numerous clinical trials, and data reported from two regimens with mean periods of castration of 55.32 days and 68.95 days, respectively, suggest that an injection interval of 6+ weeks may be a possibility. PK and PD modelling of existing data plus further clinical trials to test loading and maintenance doses will be required. 

## Figures and Tables

**Table 1:** Patient baseline characteristics (per-protocol population).

Characteristic	EP-24332T-A013	EP-24332T-A014	ARD-0301-003	ARD-0301-008	ARD-0301-010	
Administration (dose, route, schedule); *n*	90 mg SC; D0, D1, D2 *n* = 14	90 mg IM; D0, D7; *n* = 14	120 mg SC; D0, D1; *n* = 8	120 (2 × 60) mg SC; D0, D1; *n* = 8	120 (2 × 60) mg SC; D0, D1, D2; *n* = 18	180 (2 × 90) mg SC; D0, D1, D2; *n* = 20
Age (year), median (range)	71.5 (62–78)	74.0 (66–81)	69.0 (54–74)	63.5 (58–84)	70.5 (51–79)	70.5 (53–78)
Weight (kg), mean (SD)	80.93 (13.27)	76.14 (12.13)	86.38 (15.21)	88.4 (16.7)	85.7 (14.39)	82.1 (13.7)
BMI (kg/m^2^), mean (SD)	27.91 (4.41)	25.17 (3.60)	27.96 (3.28)	28.60 (4.80)	28.89 (3.69)	28.13 (4.79)
TNM grading, *n* (%)						
T (tumor)						
T2	1 (7.1)	1 (7.1)	0	0	3 (16.7)	4 (20)
T3	13 (92.9)	13 (92.9)	8 (100)	8 (100)	15 (83.3)	15 (75)
T4	0	0	0	0	0	1 (5)
N (nodes)						
N0	11 (78.6)	7 (50)	7 (87.5)	8 (100)	12 (66.7)	12 (60.0)
N1 or 2	0	0	1 (12.5)	0	1 (5.6)	2 (10)
Nx	3 (21.4)	7 (50)	0	0	5 (27.8)	6 (30)
M (metastases)						
M0	14 (100)	12 (85.7)	6 (75.0)	7 (87.5)	18 (100)	14 (70)
M1	0	2 (14.3)	2 (25.0)	1 (12.5)	0	4 (20.0)
Mx	0	0	0	0	0	2 (10.0)
Gleason score						
<6	0	0	0	0	5 (27.8)	6 (30)
6	11 (78.6)	8 (57.1)	6 (75.0)	4 (50.0)	8 (44.4)	9 (45)
7	2 (14.3)	3 (21.4)	1 (12.5)	3 (37.5)	3 (16.7)	2 (10)
8	1 (7.1)	2 (14.3)	1 12.5)	1 12.5)	0	1 (5)
9	0	1 (7.1)	0	0	2 (11.1)	2 (10)

BMI = body mass index; D = day; IM = intramuscular; SC = subcutaneous; SD = standard deviation.

**Table 2:** Mean onset and duration of castration.

Outcome	EP-24332T-A013	EP-24332T-A014	ARD-0301-003	ARD-0301-008	ARD-0301-010	
Administration (dose, route, schedule); *n*	90 mg SC; D0, D1, D2 *n* = 14	90 mg IM; D0, D7; *n* = 14	120 mg SC; D0, D1; *n* = 8	120 (2 × 60) mg SC; D0, D1; *n* = 8	120 (2 × 60) mg SC; D0, D1, D2; *n* = 18	180 (2 × 90) mg SC; D0, D1, D2; *n* = 20
Mean onset of castration (days)	1.77	2.40	1.10	1.44	1.47 ^a^	1.42
Mean sustained castration period (days)	55.32	34.77	32.45	45.85	60.00 ^a^	68.95
Castration period, range (min–max) (weeks)	4–14	3–8	0–6	0 ^b^–17	0–17	5–≥21
Castration rate at Day 28: patients with T levels <0.5 ng/mL at Day 28, *n* (%)	14 (100%)	12 (86%)	5 (63%)	4 (50%)	16 (89%)	19 (95%)
Testosterone plasma concentration at Day 28, median (range) (ng/mL)	0.09 (0.00, 0.47)	0.14 (0.00, 1.25)	0.16 (0.09, 0.26)	0.24 (0.1, 3.48)	0.20 (0.1, 3.1)	0.10 (0.1, 2.6)

D = day; IM = intramuscular; SC = subcutaneous. ^a^ Excludes 2 patients who only achieved castrate levels 3 and 2 weeks after first injection. ^b^ Two patients were only castrate at the Day 2 visit.

**Table 3:** Overview of adverse events.

Adverse Event	EP-24332T-A013	EP-24332T-A014	ARD-0301-003	ARD-0301-008	ARD-0301-010		Total *n* = 82
	90 mg SC; D0, D1, D2 *n* = 14	90 mg IM; D0, D7; *n* = 14	120 mg SC; D0, D1; *n* = 8	120 (2 × 60) mg SC; D0, D1; *n* = 8	120 (2 × 60) mg SC; D0, D1, D2; *n* = 18	180 (2 × 90) mg SC; D0, D1, D2; *n* = 20	
	# subjects (%)						
Any adverse event	9 (64.3)	0	8 (100)	8 (100)	13 (72.2)	15 (75.0)	53 (64.6)
Serious adverse event	0	0	0	0	1 (5.6)	0	1 (1.2)
Adverse events with severe intensity	0	0	0	0	0	0	0
Drug-related adverse events	7 (50.0)	0	8 (100)	8 (100)	10 (55.6)	13 (65.0)	46 (56.1)
Deaths	0	0	0	0	0	0	0
Adverse events in >1 patient in each group in the individual trials							
Injection site reaction ^a^	1 (7.1)	0	8 (100)	8 (100)	9 (50)	12 (60)	38 (46.3)
Flushing/hot flush	2 (14.3)	0	2 (25.0)	2 (25.0)	6 (33.3)	6 (30.0)	18 (22.0)
Cystitis	4 (28.6)	0	0	0	0	1 (5.0)	5 (6.1)
Influenza	0	0	0	0	3 (16.7)	1 (5.0)	4 (4.9)
Urinary retention	0	0	0	0	1 (5.6)	2 (10)	3 (3.7)
Dysuria	0	0	2 (25.0)	0	0	0	2 (2.4)

D = day; IM = intramuscular; SC = subcutaneous. ^a^ Includes all injection site AEs by preferred term, e.g., induration, bruising, reaction, erythema, hematoma and pruritus.

## Data Availability

Not applicable.

## Supplementary Materials

The following supporting information can be downloaded at: https://www.mdpi.com/article/10.3390/medicina59040681/s1, Table S1: Summary of the 5 Phase 2 clinical trials; Table S2: Pharmacokinetic data for teverelix DP; Table S3: Formal injection site inspection.
