# Supplementary material for: Safety, Pharmacokinetic and Pharmacodynamic Evaluation of Teverelix for the Treatment of Hormone-Sensitive Advanced Prostate Cancer: Phase 2 Loading-Dose-Finding Studies
Source: Medicina (Kaunas). 2023 Mar 29;59(4):681. doi: 10.3390/medicina59040681 (PMC10146264; doi:10.3390/medicina59040681)
Supplement: Supplementary file 1 [file medicina-59-00681-s001.zip › medicina-2239794-supplementary.pdf]

Supplemental Table S1. Summary of the 5 Phase 2 clinical trials

	**EP-24332T-A013**	**EP-24332T-A014**	**ARD-0301-003**	**ARD-0301-008**	**ARD-0301-010**
**EudraCT No.**	Not Applicable*	2004-001648-64	2005-002100-42	2005-005742-39	2006-004572-13
**Ethics: IEC and reference no.**	Lithuanian Bioethics Committee: 2004-03-31 No. 30/2	Lithuanian Bioethics Committee: 2004-09-01 No. 46/1	Lithuanian Bioethics Committee: 2005-07-013 No. 47	Lithuanian Bioethics Committee: 2006-01-26 No. 4	Lithuanian Bioethics Committee: 1. 2007-02-21 No. 10<br><br>IEC for investigation of drugs and pharmaceutical products (Republic of Latvia): 190916-120
Study design	Phase 2, open label study investigating the pharmacokinetics, pharmacodynamics, efficacy and safety of a loading dose regimen teverelix in patients with advanced prostate cancer				
	Multi centre	Multi centre	Single centre	Single centre	Multi centre
Administration (dose, route, schedule); n	90 mg SC; D0,1,2; n=14	90 mg IM; D0, D7; n=14	120 mg SC; D0, D1; n=8	120 (2x60) mg SC; D0, D1; n=8	120 (2x60) mg SC; D0, D1, D2; n=18<br>180 (2x90) mg SC; D0, D1, D2; n=20
Inclusion criteria	• Histologically proven adenocarcinoma of the prostate<br>• Androgen deprivation therapy suitable (advanced prostate cancer i.e. with local invasion or/and metastasis)<br>• Signed written informed consent				
Exclusion criteria	• Liver or renal function tests (ASAT/SGOT, ALAT/SGPT, total bilirubin, creatinine) exceeding twice the upper limit of the normal range, unless the elevation is attributed to hepatic metastasis<br>• Any contraindication to the use of teverelix<br>• Life expectancy of less than 1 year<br>• Baseline testosterone value below 2.31 ng/ml<br>• Bilateral orchidectomy<br>• Pre-existing hormone therapy or planned concomitant use of androgen deprivation therapy with any agent other than the investigational drug<br>• Neurological, psychiatric disease, drug or alcohol abuse which could interfere with the subject’s proper compliance<br>• Evidence of concurrent malignancy<br>• Exposure to another investigational agent within the last month<br>• Lack of ability or willingness to give informed consent<br>• Anticipated non-availability for study visits/ procedures				

	EP-24332T-A013	EP-24332T-A014	ARD-0301-003	ARD-0301-008	ARD-0301-010
Primary objective	To assess the duration of action of an initial "loading" dose regimen of Teverelix LA in terms of suppression of testosterone to below castrate level (0.5 ng/ml)				
Secondary objective	To assess:<br>• Pharmacodynamics of teverelix in terms of ability to suppress and to maintain plasma testosterone levels below castration level (< 0.5ng/ml) until (after week 3) 2 consecutive, increasing testosterone levels above castration level with the latter one above 2 ng/ml, have been recorded.<br>• Effects on Luteinizing Hormone (LH)<br>• Effects on Prostate Specific Antigen (PSA)<br>• Safety of teverelix LA in terms of :<br>o local tolerability and<br>o systemic tolerability (adverse events and changes in laboratory parameters)				

D=day; IEC=Independent Ethics Committee; IM=intramuscular; SC=subcutaneous
Testosterone 0.5 ng/ml=2 nmol/l
* Pre-dated requirement for clinical trial registration

Supplemental Table S2. Pharmacokinetic data for teverelix DP

Parameter	EP-24332T-A013	EP-24332T-A014	Parameter	ARD-0301-003	ARD-0301-008	ARD-0301-010	
Treatment	90 mg SC;<br>D0,1,2<br>n=14	90 mg IM;<br>D0, D7;<br>n=14	Treatment	120 mg SC;<br>D0, D1;<br>n=8	120 (2x60) mg SC;<br>D0, D1;<br>n=8	120 (2x60) mg SC;<br>D0, D1, D2;<br>n=18	180 (2x90) mg SC;<br>D0, D1, D2;<br>n=20
Cmax(obs) (ng/mL)	12.86 (41.2)	Dose 1: 21.5 (69.5)<br>Dose 2: 38.0 (33.8)	$Cmax_{Init1}$ (ng/mL)	13.6 (35.5)	19.6 (47.1)	17.9 (67.8)	36.6 (194.0)
AUC(0-t) (ng.h/mL)	197.0 (32.9)	Dose 1: 1734 (101.1)<br>Dose 2: 8355 (22.2)	$Cmax_{Init2}$ (ng/mL)	17.9 (27.1)	24.0 (29.2)	28.4 (112.0)	38.8 (71.0)
AUC(0-∞)	-	Dose 1: 4610	$Cmax_{Init3}$	NA	NA	30.5 (58.1)	48.2 (133.0)

Parameter	EP-24332T-A013	EP-24332T-A014	Parameter	ARD-0301-003	ARD-0301-008	ARD-0301-010	
Treatment	90 mg SC; D0,1,2 n=14	90 mg IM; D0, D7; n=14	Treatment	120 mg SC; D0, D1; n=8	120 (2x60) mg SC; D0, D1; n=8	120 (2x60) mg SC; D0, D1, D2; n=18	180 (2x90) mg SC; D0, D1, D2; n=20
(ng.h/mL)		(86.1) Dose 2: 9377 (20.0)	(ng/mL)				
AUC(all)	-	10480 (27.3)	$Cmax_{Late}$ (ng/mL)	11.1 (31.5)	9.56 (15.7)	15.09 (62.06)	23.2 (49.34)
AUC(all∞) (ng.h/mL)	-	11510 (24.6)	AUC(0-t) (Dose 1) (ng.h/mL)	189.0 (35.2)	272.7 (37.4)	209.0 (52.8)	450.0 (141.0)
T1/2elim (h)	1086.71 (593.33)	Dose 1: 166.60 (72.9) Dose 2: 404.43 (113.15)	AUC(0-t) (Dose 2) (ng.h/mL)	6331 (47.8)	7605 (21.2)	377.0 (65.2)	557.0 (55.8)
CL/F (mL/h)	-	14940 (3720)	AUC(0-t) (Dose 3) (ng.h/mL)	NA	NA	11100.0 (68.0)	16000.0 (48.8)
Tmax(obs) (h)	2.00 (0.97-9.98)	Dose 1: 2.00 (1.00, 24.00) Dose 2: 1.00 (1.00, 24.00)	AUC(all) (ng.h/mL)	6530 (47.0)	7889 (21.0)	12094.07 (57.14)	17827.78 (47.45)
			AUC(0-∞) (ng.h/mL)	8522 (28.0)	8401 (23.5)	13348.74 (58.05)	19920.18 (37.97)
			AUC(all∞) (ng.h/mL)	8747 (27.5)	8685 (22.5)	-	-
			T1/2elim (h)	351.85 (112.06)	520.33 (394.9)	512.97 (65.52)	460.13 (38.05)
			CL/F	28250 (7625)	27730 (6450)	26968.84 (58.05)	27108.19 (37.97)

Parameter	EP-24332T-A013	EP-24332T-A014	Parameter	ARD-0301-003	ARD-0301-008	ARD-0301-010	
Treatment	90 mg SC; D0,1,2 n=14	90 mg IM; D0, D7; n=14	Treatment	120 mg SC; D0, D1; n=8	120 (2x60) mg SC; D0, D1; n=8	120 (2x60) mg SC; D0, D1, D2; n=18	180 (2x90) mg SC; D0, D1, D2; n=20
			(mL/h)				
			$Tmax_{Init1}$ (h)	2.00 (1.00,4.00)	2.00 (1.00, 2.00)	2.0 (1.0, 21.2)	2.0 (1.0, 10.0)
			$Tmax_{Init2}$ (h)	2.00 (1.00,6.00)	2.00 (1.00, 6.00)	2.0 (1.0, 23.8)	4.0 (1.0, 23.9)
			$Tmax_{Init3}$ (h)	NA	NA	2.0 (1.0, 624.0)	2.0 (1.0, 456.0)
			$Tmax_{Late}$ (h)	311.87 (47.68, 551.67)	311.80 (143.87, 648.00)	336.58 (93.0, 670.0)	335.72 (94.0, 890.0)

AUC=area under the curve; CL/F=oral clearance; Cmax=maximal concentration; D=day; h=hour; IM=intramuscular; Init1, 2 or 3=relative to the first, second or third dose; NA, non-applicable; SC=subcutaneous; Tmax= time of maximum observed concentration; T1/2elim= terminal elimination half-life
Data are geometric mean (CV%) for Cmax and AUC; arithmetic mean (SD) for T1/2elim and CL/F; median (range) for Tmax

Supplemental Table S3. Formal injection site inspection

Injection site reaction	ARD-0301-003: 120 mg SC N=8		ARD-0301-008: 2x60 mg SC N=8		ARD-0301-010: 120 (2x60) mg SC N=18			ARD-0301-010: 180 (2x90) mg SC N=20		
	D0 injection site	D1 injection site	D0 injection site	D1 injection site	D0 injection site	D1 injection site	D2 injection site	D0 injection site	D1 injection site	D2 injection site
Redness	3 (37.5)	0	Injection site 1: 0 Injection site 2: 0	Injection site 1: 0 Injection site 2: 0	Injection site 1: 0 Injection site 2: 0	Injection site 1: 0 Injection site 2: 0	Injection site 1: 0 Injection site 2: 0	Injection site 1: 1 (5) Injection site 2: 2 (10)	Injection site 1: 0 Injection site 2: 0	Injection site 1: 0 Injection site 2: 0
Swelling	3 (37.5)	0	Injection site 1: 0 Injection site 2: 0	Injection site 1: 0 Injection site 2: 0	Injection site 1: 0 Injection site 2: 0	Injection site 1: 0 Injection site 2: 0	Injection site 1: 0 Injection site 2: 0	Injection site 1: 1 (5) Injection site 2: 0	Injection site 1: 0 Injection site 2: 0	Injection site 1: 0 Injection site 2: 0
Induration	6 (75.0)	5 (62.5)	Injection site 1: 8 (100%) Injection site 2: 8 (100%)	Injection site 1: 8 (100%) Injection site 2: 8 (100%)	Injection site 1: 8 (44.4) Injection site 2: 8 (44.4)	Injection site 1: 8 (44.4) Injection site 2: 8 (44.4)	Injection site 1: 9 (50) Injection site 2: 9 (50)	Injection site 1: 11 (55) Injection site 2: 9 (45)	Injection site 1: 9 (45) Injection site 2: 9 (45)	Injection site 1: 8 (40) Injection site 2: 8 (40)
Pain	0	0	Injection site 1: 6 (75%) (mild) Injection site 2: : 6 (75%) (mild)	Injection site 1: 2 (25%) (mild) Injection site 2: 2 (25%) (mild)	Injection site 1: 1 (5.6) (mild) Injection site 2: 1 (5.6) (mild)	Injection site 1: 0 Injection site 2: 0	Injection site 1: 0 Injection site 2: 0	Injection site 1: 3 (15) (mild) Injection site 2: 3 (15) (mild)	Injection site 1: 0 Injection site 2: 0	Injection site 1: 0 Injection site 2: 1 (5)

Injection site reaction	ARD-0301-003: 120 mg SC<br>N=8		ARD-0301-008: 2x60 mg SC<br>N=8		ARD-0301-010: 120 (2x60) mg SC<br>N=18			ARD-0301-010: 180 (2x90) mg SC<br>N=20		
	D0 injection site	D1 injection site	D0 injection site	D1 injection site	D0 injection site	D1 injection site	D2 injection site	D0 injection site	D1 injection site	D2 injection site
Itching	0	0	Injection site 1: 0<br>Injection site 2: 0	Injection site 1: 0<br>Injection site 2: 0	0	0	0	0	Injection site 1: 1 (5)<br>Injection site 2: 2 (10)	0

D=day; IM=intramuscular; SC=subcutaneous
